# Causal associations between gut microbiota, gut microbiota-derived metabolites, and cerebrovascular diseases: a multivariable Mendelian randomization study

**DOI:** 10.3389/fcimb.2023.1269414

**Published:** 2023-11-08

**Authors:** Dihui Lin, Yingjie Zhu, Zhi Tian, Yong Tian, Chengcai Liang, Xiaowei Peng, Jinping Li, Xinrui Wu

**Affiliations:** ^1^ School of Medicine, Jishou University, Jishou, China; ^2^ Department of Neurosurgery, The First Affiliated Hospital of Jishou University, Jishou, China; ^3^ Department of Neurology, The First Affiliated Hospital of Jishou University, Jishou, China; ^4^ Department of Orthopedics, The Affiliated Changsha Central Hospital, Changsha, China

**Keywords:** Mendelian randomization, gut microbiota, cerebrovascular diseases, gut microbiota-derived metabolites, causal association

## Abstract

**Background:**

Mounting evidence has demonstrated the associations between gut microbiota, gut microbiota-derived metabolites, and cerebrovascular diseases (CVDs). The major categories of CVD are ischemic stroke (IS), intracerebral hemorrhage (ICH), and subarachnoid hemorrhage (SAH). However, the causal relationship is still unclear.

**Methods:**

A two-sample Mendelian randomization (MR) study was conducted leveraging the summary data from genome-wide association studies. The inverse variance-weighted, maximum likelihood, weighted median, and MR.RAPS methods were performed to detect the causal relationship. Several sensitivity analyses were carried out to evaluate potential horizontal pleiotropy and heterogeneity. Finally, reverse MR analysis was conducted to examine the likelihood of reverse causality, and multivariable MR was performed to adjust the potential confounders.

**Results:**

We collected 1,505 host single nucleotide polymorphisms (SNPs) linked to 119 gut microbiota traits and 1,873 host SNPs associated with 81 gut metabolite traits as exposure data. Among these, three gut bacteria indicated an elevated risk of IS, two of ICH, and one of SAH. In contrast, five gut bacteria were associated with a reduced risk of IS, one with ICH, and one with SAH. Our study also demonstrated the potential causal associations between 11 gut microbiota-derived metabolites and CVD.

**Conclusions:**

This study provided evidence of the causal relationship between gut microbiota, gut microbiota-derived metabolites, and CVD, thereby offering novel perspectives on gut biomarkers and targeted prevention and treatment for CVD.

## Introduction

Cerebrovascular diseases (CVDs) are characterized by pathological changes in cerebral blood vessels resulting in brain dysfunction, such as hemiplegia and language disorder (World Health Organization, 2021). The major categories are ischemic stroke (IS), intracerebral hemorrhage (ICH), and subarachnoid hemorrhage (SAH). Despite years of prevention and treatment, CVD continues to be the second leading cause of mortality and disability globally (DALYs, G.B.D., and Collaborators, H, 2016; Mortality, G.B.D., and Causes of Death, C, 2016). With the aging of the population, the prevalence of CVD increases annually, which poses a substantial hazard to human life and places a significant burden on healthcare systems (Collaborators, G.B.D.C.o.D, 2017; Feigin et al., 2017). Nevertheless, the exact pathogenesis of CVD, as well as effective strategies for its prevention and treatment, remained uncertain.

Gut microbiota is a complex bacterial community that resides in the intestine. A growing body of research suggests that the gut microbiota and microbiota-derived metabolites play a significant role in CVD through the microbiota–gut–brain axis (Peh et al., 2022; Zou et al., 2022). Dysbiosis of the gut microbiome leads to increased gut permeability and activation of the intestinal immune system, allowing it to penetrate brain tissue via the blood–brain barrier (Hu et al., 2022). Clinical cross-sectional studies have identified gut microbiota disorders in CVD patients (Huang et al., 2019; Li et al., 2020; Ling et al., 2020b). It is reported that *Firmicutes* displayed a growing trend, but *Bacteroidetes* had a declining tendency in stroke patients (Singh et al., 2016). However, existing studies have produced inconsistent results. For instance, Yin et al. observed a significant decrease in the abundance of *Bacteroides* and *Prevotella* in IS patients compared with healthy controls (Yin et al., 2015), while another small cross-sectional study (*N* = 10) reported the opposite results (Wang et al., 2018). The gut microbiota-derived metabolites are key actors in host–microbiota crosstalk, which influences the host’s brain function and behavior, such as short-chain fatty acids (SCFAs) (Liu et al., 2020), trimethylamine N-oxide (TMAO) (Schiattarella et al., 2017), and butyrate (Haak et al., 2021). However, most evidence came from observational studies, making it difficult to determine the temporal association between exposure and outcome. Furthermore, confounding factors of gut microbiota–CVD-related studies were difficult to measure and control due to the complicated environment of the intestine and cerebral blood vessels.

Mendelian randomization (MR) analysis integrates data from genome-wide association studies (GWAS) and utilizes genetic variations as instrumental variables (IVs) to assess the causal relationship between exposures and outcomes (Greenland, 2000). The MR approach should conform to three fundamental assumptions: 1) IVs should be highly associated with exposure, 2) IVs should be independent of confounders that affect exposure and outcome, and 3) IVs should affect the outcome only through exposure. With the advantages of controlling confounders and eliminating reverse causality, MR analysis has been extensively utilized to investigate the causality between gut microbiota and human complex diseases like T2DM (Sanna et al., 2019), chronic kidney disease (Xu et al., 2020), and Alzheimer’s disease (Hughes et al., 2020). In the study, using summary data from the most extensive and up-to-date GWAS datasets, we employed the MR method to investigate the causal relationship between gut microbiota, gut microbiota-derived metabolites, and CVD.

## Materials and methods

### Data sources

Summary data for gut microbiota serving as exposure were obtained from a multi-ethnic GWAS, which includes 18,340 individuals (Kurilshikov et al., 2021). 16S rRNA gene sequencing profiles and genotyping data were coordinated to perform the genome-wide meta-analysis of the association between human genetic variants and the gut microbiota. Employing SILVA as the reference database (Quast et al., 2013), all the data were annotated to genus and higher levels.

Pooled data for gut microbiota-derived metabolites were obtained from the most comprehensive metabolite GWAS (Shin et al., 2014) so far, which was carried out among people of European ancestry (Twins UK and KORA cohort, *N* = 7,824). The GWAS examined 486 human metabolites obtained from both cohorts at each single nucleotide polymorphism (SNP). Then, we manually searched the HMDB database (Wishart et al., 2018) to screen out human gut microbiota-derived metabolites.

The MEGASTROKE consortium provided genetic variations for IS including 34,217 cases and 406,111 controls (Malik et al., 2018). Summary data for ICH and SAH were obtained from the FinnGen consortium data (Kurki et al., 2022), which included 202,833 subjects (1,687 cases/201,146 controls) and 16,381,733 subjects (1,338 cases/16,380,395 controls), respectively. Genotyped by the Illumina platform and Affymetrix chip arrays, association studies were performed with sex, age, genotyping batch, and 10 main components adjusted.

All GWAS datasets were publicly available with limited sample overlap and already obtained ethical approvals in original studies. Details of GWAS utilized in this study are shown in **Supplementary Table 1**.

### Selection of instrumental variables

In order to select optimal instrumental variables (IVs), several quality control steps were conducted. First, IVs were selected from SNPs associated with gut microbiota and gut microbiota-derived metabolites at the locus-wide significance level (*P*< 1 × 10^−5^) (Benjamini and Hochberg, 1995). Second, the PLINK clumping method on linkage disequilibrium (LD) with *r^2^* <0.001 and a clumping window of 10,000 kb was performed to identify the lead SNPs. Third, we calculated each exposure’s *F*-statistic using the formula $F=\frac{R^{2}\left(n-1-k\right)}{\left(1-R^{2}\right)k}$ , where *R* ^2^, *n*, and *k* mean the estimated exposure variance explained by IVs, sample size, and the number of IVs, respectively (Palmer et al., 2012). If the *F*-statistics were below 10, the IVs would be excluded to prevent weak IVs’ bias (Staiger and Stock, 1997).

### Statistical analyses

To estimate the causality between gut microbiota, gut microbiota-derived metabolites, and CVD, the two-sample MR analysis was performed. The inverse variance-weighted (IVW) method was performed as the primary MR analysis, which is a meta-analysis method that combines the Wald ratio estimates of each IV and restricts the intercept to zero. If there is no horizontal pleiotropy, results from IVW would be unbiased (Burgess et al., 2016). Effect estimates for causal associations were reported in odds ratios with 95% confidence intervals for binary outcomes (Dekking, 2007; Szumilas, 2010). In addition, *P*-values were adjusted for several comparisons at the significance level (*q*-value< 0.1) by false discovery rate (FDR) (Storey and Tibshirani, 2003). If exposures with *P*<0.05 while FDR corrected *q*-value >0.1, they were reported as potentially causal associations. Other sensitivity analyses were carried out to ensure the reliability of the results, including maximum likelihood (Thompson et al., 2005), weighted median (Bowden et al., 2016), and MR robust adjusted profile score (MR.RAPS) (Zhao et al., 2020). When heterogeneity and horizontal pleiotropy are assumed to be absent, the maximum likelihood method is comparable to IVW with smaller standard errors and more unbiased results (Pierce and Burgess, 2013). The weighted median method can obtain robust assumptions when less than 50% of SNPs are invalid (Bowden et al., 2016). MR.RAPS offers robust estimates to correct for systematic and idiosyncratic pleiotropy (Zhao et al., 2020).

Cochran *Q* statistic was performed to examine the heterogeneity, and the leave-one-out sensitivity analysis was employed to examine if each SNP was accountable for the causative outcomes (Hemani et al., 2017). Moreover, the MR-Egger intercept and the Mendelian randomization pleiotropy residual sum and outlier (MR-PRESSO) global test were applied to detect the potential horizontal pleiotropy between IVs and the outcome (Hemani et al., 2017).

Reverse MR analysis was also conducted to investigate the reverse causality between CVD and gut microbiota as well as gut microbiota-derived metabolites, which used the same setting and datasets as in the forward MR analysis except for changing the original exposure to outcome. Finally, we performed a multivariable MR (MVMR) analysis to distinguish each confounder’s direct influence (Burgess and Thompson, 2015). Three confounders, smoking (IEU number: ieu-b-4877), alcohol drinking (IEU number: ukb-b-5779), and hypertension (IEU number: ukb-a-61), were considered for the MVMR analysis by using the IVW method (Burgess and Thompson, 2015).

**Figure 1:**
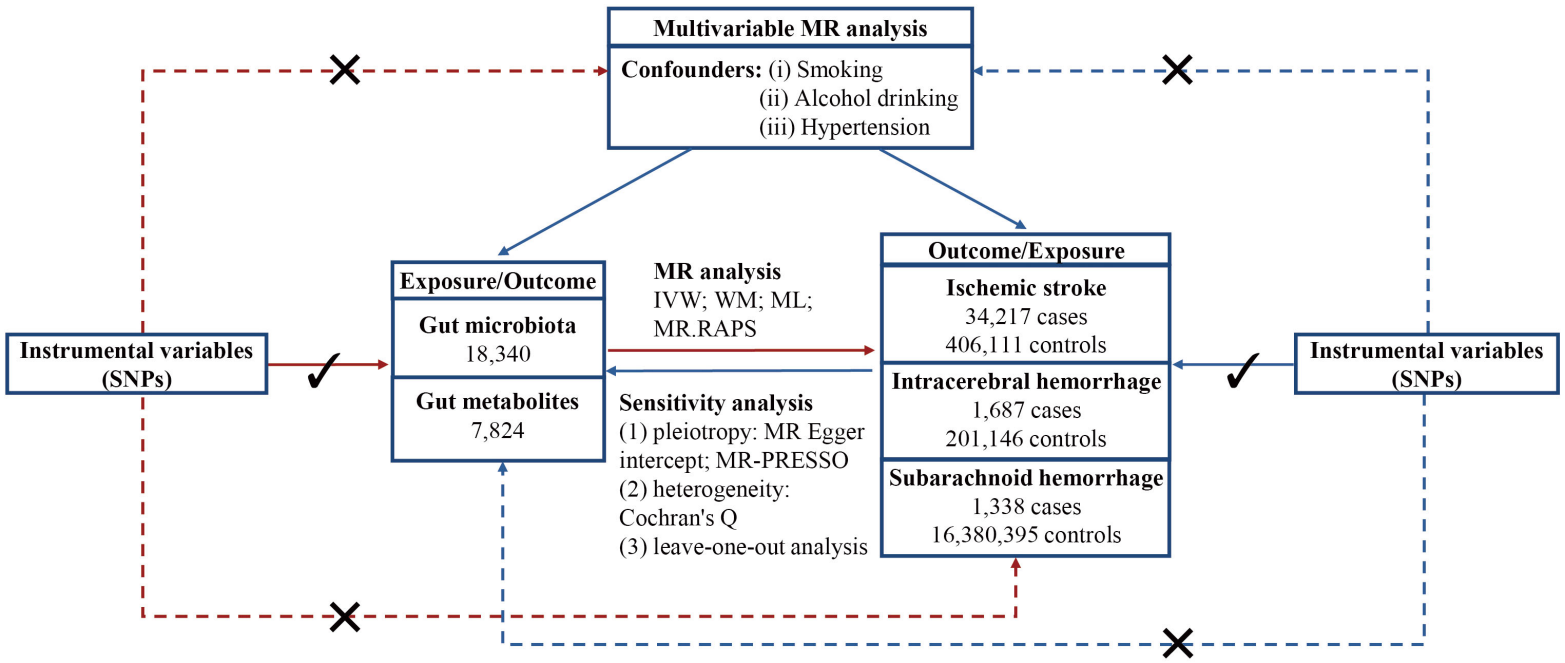
Flowchart of this study. MR, Mendelian randomization; SNP, single nucleotide polymorphism; IVW, inverse variance weighted; WM, weighted mean; ML, maximum likelihood; MR.RAPS, Mendelian randomization robust adjusted profile score; MR-PRESSO, Mendelian randomization pleiotropy residual sum and outlier.

The flowchart of this study is displayed in **Figure 1**. All statistical analyses were performed using “TwoSampleMR,” “MRPRESSO,” and “*q*value” packages in R software.

## Results

A total of 1,505 lead SNPs associated with 119 bacterial genera were included for gut microbiota, and 1,873 lead SNPs associated with 81 traits were identified for gut microbiota-derived metabolites. The characters of the selected IVs are presented in **Supplementary Tables 2**-**5**.

### Association between gut microbiota and cerebrovascular diseases

**Figure 2:**
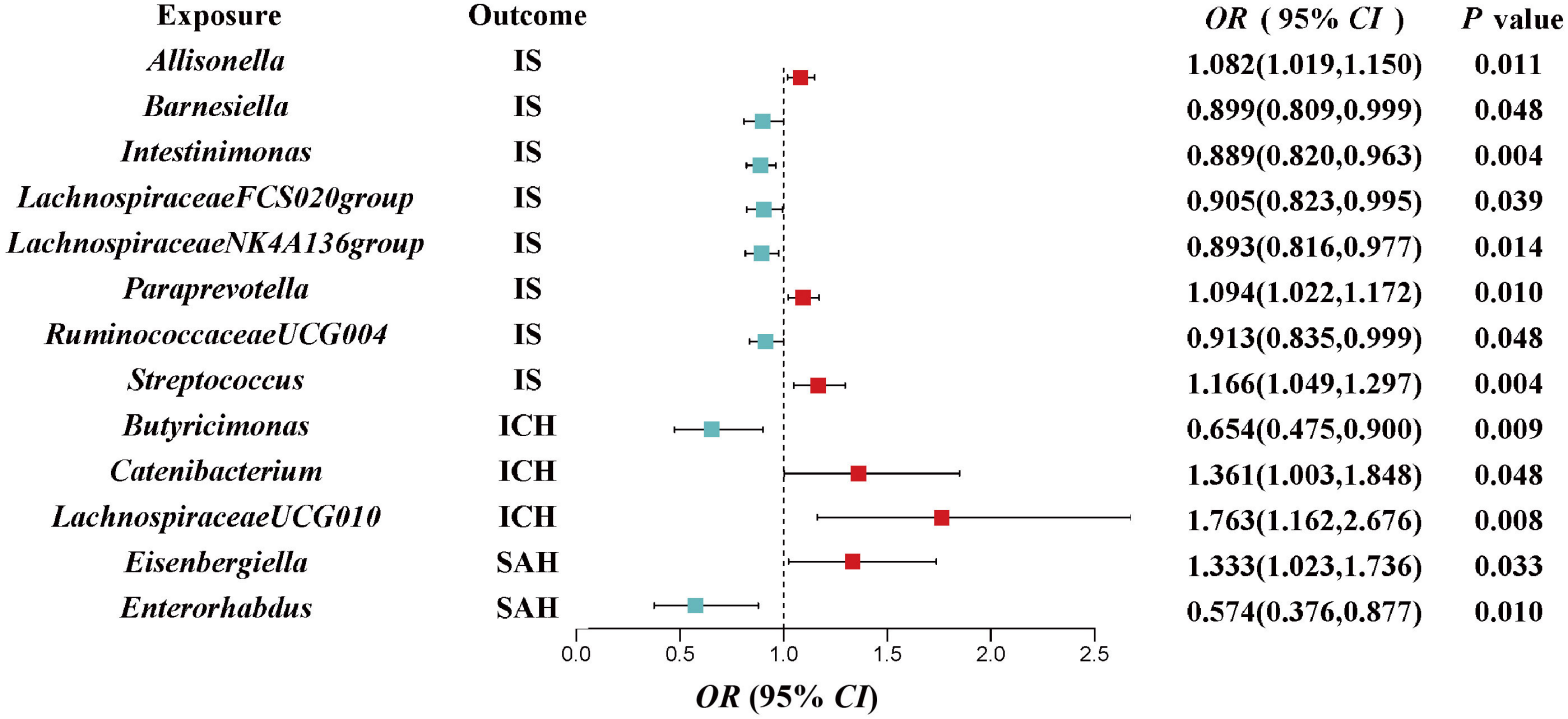
Associations of genetically predicted gut microbiota with risk of CVD using the IVW method. The colored block represents the point estimation of OR and the horizontal black line represents the 95% CI of OR. CVD, cerebrovascular disease; IVW, inverse variance weighted; IS, ischemic stroke; ICH, intracerebral hemorrhage; SAH, subarachnoid hemorrhage; OR, odds ratio; CI, confidence interval.

The IVW estimations showing higher genetically predicted *Allisonella* (OR, 1.08; 95% CI, 1.019–1.150; *P* = 0.011), *Paraprevotella* (OR, 1.094; 95% CI, 1.022–1.172; *P* = 0.010), and *Streptococcus* (OR, 1.166; 95% CI, 1.049–1.297; *P* = 0.004) were associated with a higher risk of IS, while genetically increased *Barnesiella* (OR, 0.899; 95% CI, 0.809–0.999; *P* = 0.048), *Intestinimonas* (OR, 0.889; 95% CI, 0.820–0.963; *P* = 0.004), *LachnospiraceaeFCS020group* (OR, 0.905; 95% CI, 0.823–0.995; *P* = 0.039), *LachnospiraceaeNK4A136group* (OR, 0.893; 95% CI, 0.816–0.977; *P* = 0.014), and *RuminococcaceaeUCG004* (OR, 0.913; 95% CI, 0.835–0.999; *P* = 0.048) were associated with a lower risk of IS. However, there is no significant causal association after *q*-value adjustment (**Figure 2**).

We also investigated the potential causality between gut microbiota and ICH as well as SAH, using the same methods, respectively. Our results indicated that genetically higher abundances of *Catenibacterium* (OR, 1.361; 95% CI, 1.003–1.848; *P* = 0.0423) and *LachnospiraceaeUCG010* (OR, 1.763; 95% CI, 1.162–2.676; *P* = 0.008) were associated with a higher risk of ICH, but higher abundances of *Butyricimonas* (OR, 0.654; 95% CI, 0.475–0.9; *P* = 0.009) were associated with a protective effect against ICH. Additionally, an increase in *Eisenbergiella* (OR, 1.333; 95% CI, 1.023–1.736; *P* = 0.033) was related to a greater risk of SAH, but an increase in *Enterorhabdus* (OR, 0.574; 95% CI, 0.376–0.877; *P* = 0.01) was associated with a decreased probability of SAH (**Figure 2**).

**Figure 3:**
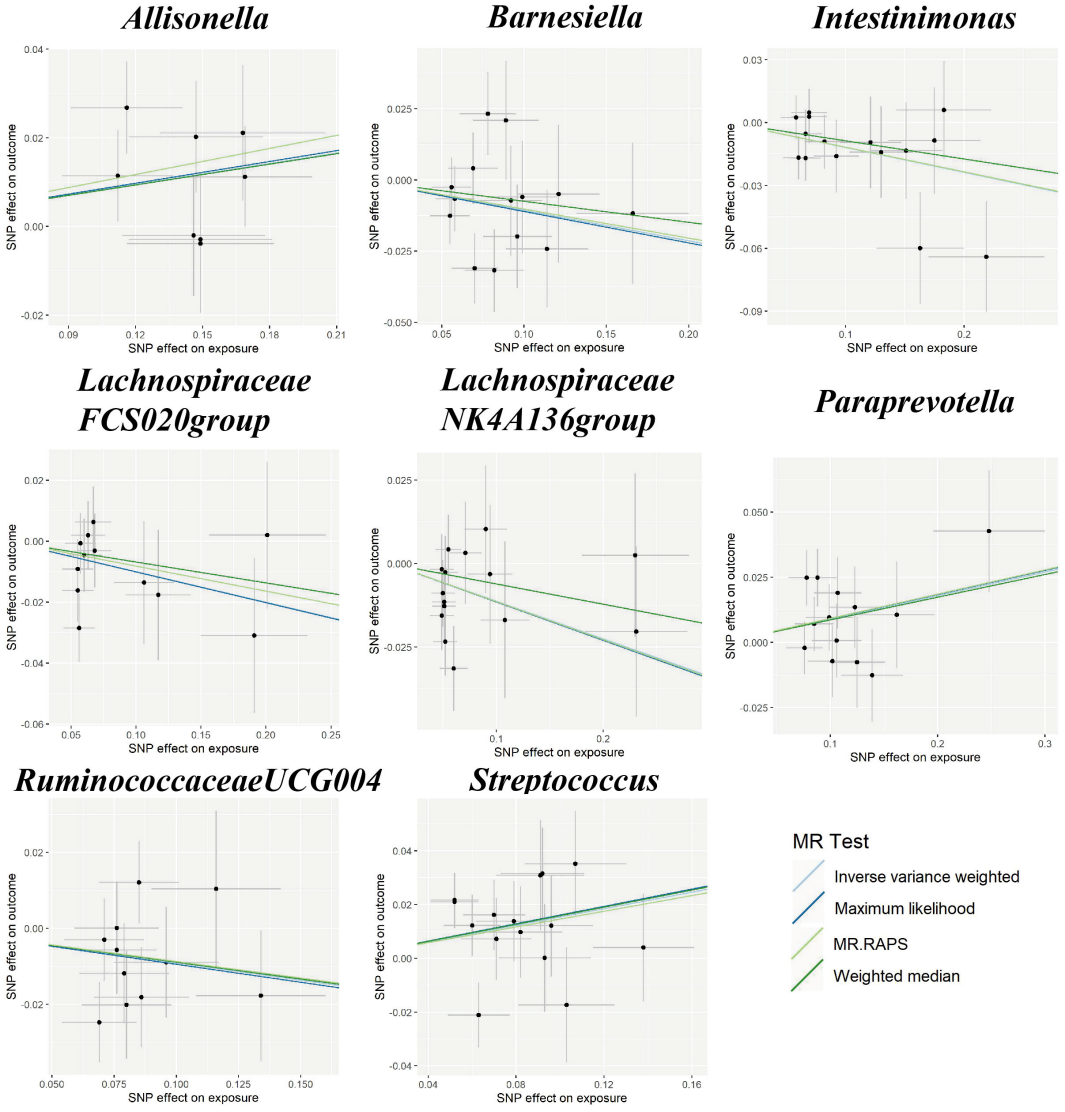
Scatter plots for the causal association between gut microbiota and IS. SNP effects were plotted into lines for the inverse variance-weighted test (light blue line), maximum likelihood (dark blue line), MR.RAPS (light green line), and weighted median (dark green line). The slope of the line corresponded to the causal estimation. IS, ischemic stroke; MR.RAPS, Mendelian randomization robust adjusted profile score.

**Figure 4:**
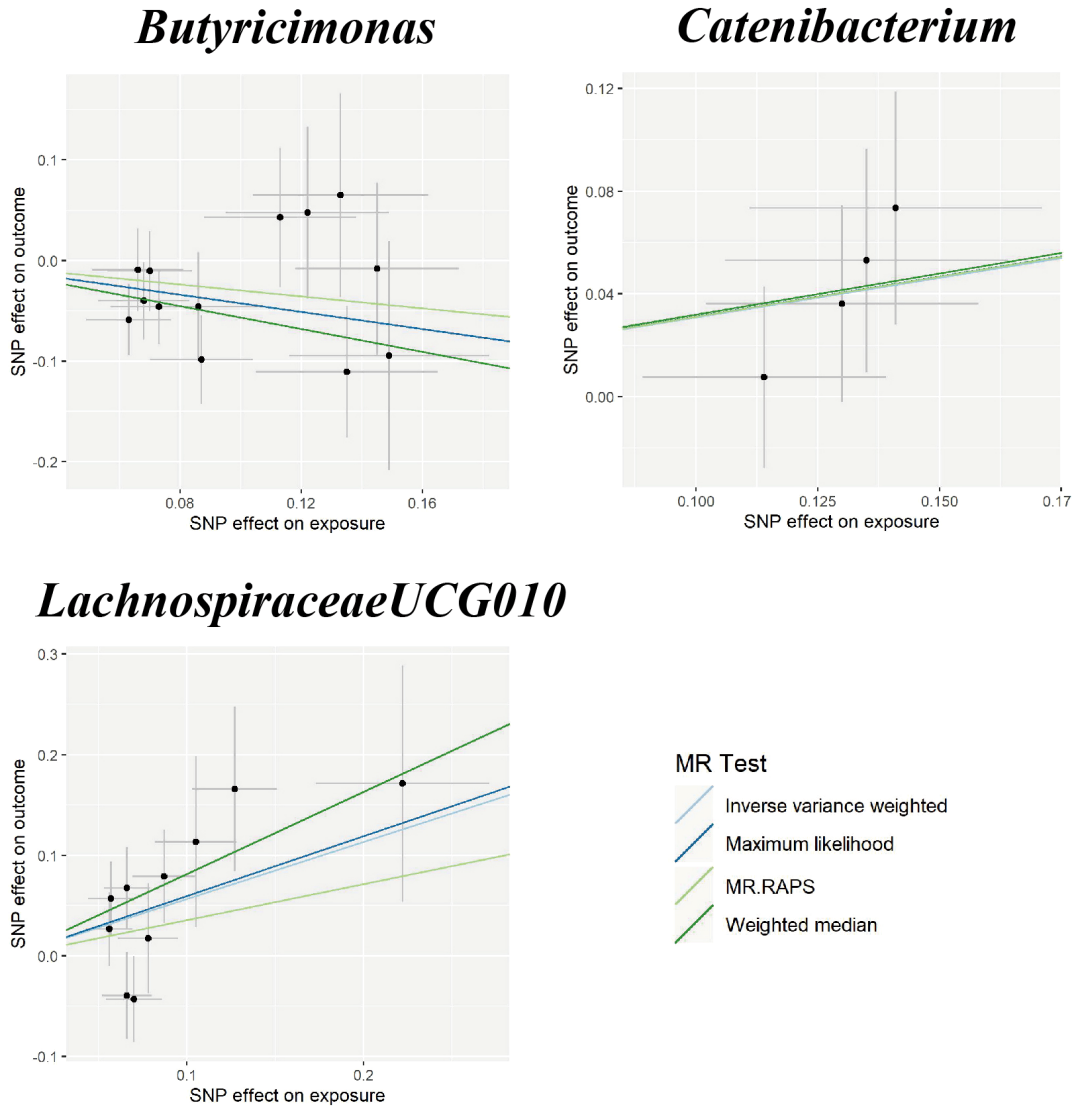
Scatter plots for the causal association between gut microbiota and ICH. SNP effects were plotted into lines for the inverse variance-weighted test (light blue line), maximum likelihood (dark blue line), MR.RAPS (light green line), and weighted median (dark green line). The slope of the line corresponded to the causal estimation. ICH, intracerebral hemorrhage; MR.RAPS, Mendelian randomization robust adjusted profile score.

**Figure 5:**
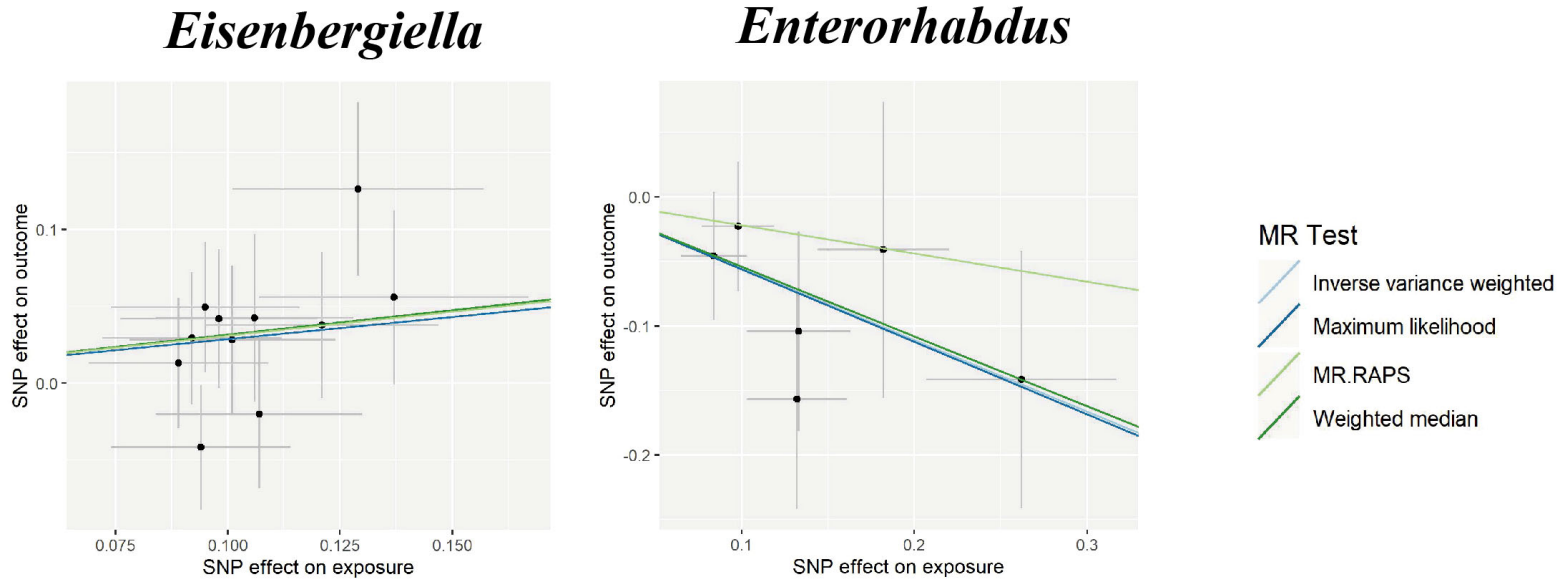
Scatter plots for the causal association between gut microbiota and SAH. SNP effects were plotted into lines for the inverse variance-weighted test (light blue line), maximum likelihood (dark blue line), MR.RAPS (light green line), and weighted median (dark green line). The slope of the line corresponded to the causal estimation. SAH, subarachnoid hemorrhage; MR.RAPS, Mendelian randomization robust adjusted profile score.

In the sensitivity analyses, the association between gut microbiota and CVD remained consistent (**Table 1**, **Figures 3**–**5**). *F*-statistics varied from 18.41 to 59.85, indicating that no weak IV bias was observed. Additionally, Cochran *Q* statistic showed no notable heterogeneity, and the leave-one-out analysis indicated that the causal relationship cannot be driven by any single SNP. Moreover, the results of MR-PRESSO suggested that there was no significant horizontal pleiotropy (**Supplementary Table 6**, **Supplementary Figures 1**-**3**).

**Table 1:** MR analyses of gut microbiota on CVD by different methods.

Exposure	Outcome	*F* statistics	Inverse variance weighted		Maximum likelihood		Weighted median		MR.RAPS	
			OR (95% CI)	*P*	OR (95% CI)	*P*	OR (95% CI)	*P*	OR (95% CI)	*P*
*Allisonella*	IS	25.49	1.082 (1.019, 1.150)	0.011	1.085 (1.019, 1.155)	0.011	1.081 (0.996, 1.174)	0.063	1.103 (1.037, 1.174)	0.002
*Barnesiella*	IS	24.81	0.899 (0.809, 0.999)	0.048	0.896 (0.810, 0.991)	0.032	0.929 (0.806, 1.070)	0.304	0.902 (0.821, 0.991)	0.030
*Intestinimonas*	IS	26.20	0.889 (0.820, 0.963)	0.004	0.890 (0.819, 0.966)	0.006	0.917 (0.821, 1.024)	0.124	0.889 (0.822, 0.963)	0.004
*LachnospiraceaeFCS020group*	IS	29.58	0.905 (0.823, 0.995)	0.039	0.904 (0.820, 0.997)	0.044	0.934 (0.824, 1.059)	0.287	0.981 (0.898, 1.073)	0.072
*LachnospiraceaeNK4A136group*	IS	23.66	0.893 (0.816, 0.977)	0.014	0.891 (0.811, 0.980)	0.017	0.941 (0.829, 1.068)	0.348	0.892 (0.812, 0.980)	0.017
*Paraprevotella*	IS	22.92	1.094 (1.022, 1.172)	0.010	1.097 (1.022, 1.176)	0.010	1.091 (0.991, 1.201)	0.076	1.098 (1.022, 1.181)	0.036
*RuminococcaceaeUCG004*	IS	23.66	0.913 (0.835, 0.999)	0.048	0.910 (0.830, 0.998)	0.045	0.915 (0.808, 1.037)	0.164	0.917 (0.837, 1.005)	0.063
*Streptococcus*	IS	23.66	1.166 (1.049, 1.297)	0.004	1.175 (1.062, 1.300)	0.002	1.172 (1.021, 1.346)	0.024	1.157 (1.048, 1.277)	0.004
*Butyricimonas*	ICH	28.77	0.654 (0.475, 0.900)	0.009	0.653 (0.472, 0.904)	0.010	0.567 (0.370, 0.868)	0.009	1.109 (0.355, 3.464)	0.045
*Catenibacterium*	ICH	18.41	1.361 (1.003, 1.848)	0.048	1.367 (0.997, 1.874)	0.052	1.377 (0.957, 1.981)	0.085	10.086 (0.227, 447.877)	0.066
*LachnospiraceaeUCG010*	ICH	26.02	1.763 (1.162, 2.676)	0.008	1.814 (1.207, 2.724)	0.004	2.262 (1.341, 3.817)	0.002	3.248 (0.914, 11.546)	0.106
*Eisenbergiella*	SAH	23.66	1.333 (1.023, 1.736)	0.033	1.334 (1.018, 1.749)	0.037	1.374 (0.979, 1.930)	0.067	1.365 (1.046, 1.781)	0.022
*Enterorhabdus*	SAH	33.98	0.574 (0.376, 0.877)	0.010	0.570 (0.367, 0.886)	0.012	0.582 (0.339, 1.000)	0.050	0.803 (0.568, 1.135)	0.214

MR, Mendelian randomization; CVD, cerebrovascular disease; OR, odds ratio; CI, confidence interval; MR.RAPS, Mendelian randomization robust adjusted profile score; IS, ischemic stroke; ICH, intracerebral hemorrhage; SAH, subarachnoid hemorrhage.

Nevertheless, the results of reverse MR did not reveal any correlations between CVD and gut microbiota (**Supplementary Table 7**). Detailed results of the sensitivity analyses are listed in **Supplementary Table 8**. We conducted an MVMR analysis to examine the cause–effect of gut microbiota on CVD after adjustment of three confounding factors (smoking, alcohol drinking, and hypertension). For the genus *Streptococcus*, after adjusting for alcohol drinking (OR = 1.182; 95% CI, 1.070–1.306; *P* = 0.001), smoking (OR = 1.189; 95% CI, 1.045–1.354; *P* = 0.009), and hypertension (OR = 1.168; 95% CI, 1.052–1.298; *P* = 0.004), the *Streptococcus* remained causally associated with CVD. We found that other gut microbiota continued to be causally related to CVD and had a more substantial impact than the causal effect found by univariable MR (**Table 2**).

**Table 2:** Multivariable MR analyses of gut microbiota on CVD after adjusting confounding factors.

Exposure	Outcome	Alcohol drinking		Smoking		Hypertension	
		OR (95% CI)	*P*	OR (95% CI)	*P*	OR (95% CI)	*P*
*Allisonella*	IS	1.069 (1.003, 1.139)	0.040	1.098 (1.014, 1.189)	0.021	1.059 (1.007, 1.113)	0.025
*Barnesiella*	IS	0.887 (0.785, 1.001)	0.052	0.873 (0.762, 0.999)	0.048	0.876 (0.777, 0.988)	0.031
*Intestinimonas*	IS	0.909 (0.843, 0.980)	0.013	0.892 (0.831, 0.958)	0.002	0.894 (0.823, 0.971)	0.008
*LachnospiraceaeFCS020group*	IS	0.907 (0.833, 0.987)	0.024	0.955 (0.880, 1.036)	0.265	0.932 (0.868, 1.000)	0.052
*LachnospiraceaeNK4A136group*	IS	0.884 (0.806, 0.968)	0.008	0.897 (0.807, 0.998)	0.047	0.949 (0.824, 1.094)	0.473
*Paraprevotella*	IS	1.087 (0.994, 1.188)	0.069	1.109 (1.032, 1.191)	0.005	1.097 (1.002, 1.201)	0.046
*RuminococcaceaeUCG004*	IS	0.893 (0.796, 1.002)	0.055	0.908 (0.807, 1.021)	0.107	0.907 (0.852, 0.966)	0.002
*Streptococcus*	IS	1.182 (1.070, 1.306)	0.001	1.189 (1.045, 1.354)	0.009	1.168 (1.052, 1.298)	0.004
*Butyricimonas*	ICH	0.587 (0.416, 0.828)	0.002	0.545 (0.325, 0.915)	0.022	0.545 (0.329, 0.905)	0.019
*Catenibacterium*	ICH	1.577 (1.394, 1.785)	5.02E−13	1.459 (0.967, 2.201)	0.072	1.301 (1.051, 1.610)	0.015
*LachnospiraceaeUCG010*	ICH	1.609 (1.153, 2.244)	0.005	1.659 (1.104, 2.494)	0.015	1.759 (1.137, 2.722)	0.011
*Eisenbergiella*	SAH	1.332 (1.053, 1.685)	0.017	1.352 (1.065, 1.717)	0.013	1.300 (1.008, 1.677)	0.043
*Enterorhabdus*	SAH	0.579 (0.378, 0.888)	0.012	0.578 (0.463, 0.721)	1.15E−06	0.619 (0.485, 0.790)	1.21E−04

Multivariable MR (MVMR) analyses were conducted by using the inverse variance-weighted (IVW) method.

MR, Mendelian randomization; CVD, cerebrovascular disease; OR, odds ratio; CI, confidence interval; IS, ischemic stroke; ICH, intracerebral hemorrhage; SAH, subarachnoid hemorrhage.

### Association between gut metabolites and cerebrovascular diseases

We obtained 11 suggestive estimates of the effects of gut metabolites on CVD among the 81 gut microbiota-derived metabolites that were included in our study. Increased abundances of cholesterol (OR, 1.639; 95% CI, 1.053–2.55; *P* = 0.028), leucine (OR, 1.548; 95% CI, 1.04–2.303; *P* = 0.031), and taurodeoxycholate (OR, 1.138; 95% CI, 1.005–1.289; *P* = 0.041) were found to be associated with a higher risk of IS, while increased glycerate (OR, 0.562; 95% CI, 0.319–0.993; *P* = 0.047), indolelactate (OR, 0.608; 95% CI, 0.396–0.936; *P* = 0.024), ornithine (OR, 0.384; 95% CI, 0.172–0.857; *P* = 0.019), and threonate (OR, 0.783; 95% CI, 0.617–0.994; *P* = 0.044) were protective factors for IS. Furthermore, 7α-hydroxy-3-oxo-4-cholestenoate (7-Hoca) (OR, 0.091; 95% CI, 0.013–0.659; *P* = 0.018), choline (OR, 0.02; 95% CI, 0.001–0.367; *P* = 0.008), and glycine (OR, 0.343; 95% CI, 0.147–0.8; *P* = 0.013) were revealed as protective factors of SAH, while tyrosine (OR, 5.908; 95% CI, 1.178–29.641; *P* = 0.099) was demonstrated to enhance the risk of ICH (**Table 3**).

**Table 3:** MR analyses of gut microbiota-derived metabolites on CVD by the IVW method.

Exposure	Outcome	*F* statistics	OR (95% CI)	*P*	*q*-value
Cholesterol	IS	29.07	1.639 (1.053, 2.550)	0.028	0.320
Glycerate	IS	29.59	0.562 (0.319, 0.993)	0.047	0.320
Indolelactate	IS	27.40	0.608 (0.396, 0.936)	0.024	0.320
Leucine	IS	56.91	1.548 (1.040, 2.303)	0.031	0.320
Ornithine	IS	40.09	0.384 (0.172, 0.857)	0.019	0.320
Taurodeoxycholate	IS	29.18	1.138 (1.005, 1.289)	0.041	0.320
Threonate	IS	21.86	0.783 (0.617, 0.994)	0.044	0.320
Tyrosine	ICH	27.88	5.908 (1.178, 29.641)	0.031	0.990
7-Hoca	SAH	30.36	0.091 (0.013, 0.659)	0.018	0.476
Choline	SAH	32.14	0.020 (0.001, 0.367)	0.008	0.476
Glycine	SAH	59.85	0.343 (0.147, 0.800)	0.013	0.476

MR, Mendelian randomization; CVD, cerebrovascular disease; IVW, inverse variance weighted; OR, odds ratio; CI, confidence interval; IS, ischemic stroke; ICH, intracerebral hemorrhage; SAH, subarachnoid hemorrhage.

Additionally, following the sensitivity analyses, those aforementioned results were deemed to be trustworthy without pleiotropy (**Table 4**, **Supplementary Table 6**, and **Supplementary Figures 4**-**6**), but we did not find a significant association after the *q*-value adjustment. Reverse MR analysis demonstrated that CVD had no causal association with gut microbiota-derived metabolites except for the association between IS and cholesterol, leucine, and taurodeoxycholate (**Supplementary Tables 7**, **8**). We conducted an MVMR analysis to examine the causality of gut microbiota-derived metabolites on CVD after adjusting for confounding factors. For the protective factor ornithine, after adjusting for alcohol drinking (OR = 0.547; 95% CI, 0.326–0.916; *P* = 0.022), smoking (OR = 0.527; 95% CI, 0.348–0.798; *P* = 0.002), and hypertension (OR = 0.548; 95% CI, 0.311–0.967; *P* = 0.038), we found it continued to be causally associated with CVD and had a more significant impact than the causal relationship found by univariable MR (**Table 5**).

**Table 4:** MR analyses of gut microbiota-derived metabolites on CVD by different methods.

Exposure	Outcome	Inverse variance weighted		Maximum likelihood		Weighted median		MR.RAPS	
		OR (95% CI)	*P*	OR (95% CI)	*P*	OR (95% CI)	*P*	*OR* (95%*CI*)	*P*
Cholesterol	IS	1.639 (1.053, 2.550)	0.028	1.676 (1.135, 2.474)	0.009	1.768 (0.994, 3.148)	0.053	1.686 (1.151, 2.472)	0.007
Glycerate	IS	0.562 (0.319, 0.993)	0.047	0.544 (0.313, 0.945)	0.031	0.507 (0.243, 1.057)	0.070	0.497 (0.312, 0.793)	0.003
Indolelactate	IS	0.608 (0.396, 0.936)	0.024	0.618 (0.431, 0.886)	0.009	0.604 (0.371, 0.986)	0.044	0.562 (0.397, 0.797)	0.001
Leucine	IS	1.548 (1.040, 2.303)	0.031	1.579 (1.100, 2.266)	0.013	1.188 (0.667, 2.115)	0.559	1.527 (1.080, 2.158)	0.017
Ornithine	IS	0.384 (0.172, 0.857)	0.019	0.347 (0.155, 0.777)	0.010	0.484 (0.187, 1.249)	0.133	0.593 (0.334, 1.054)	0.075
Taurodeoxycholate	IS	1.138 (1.005, 1.289)	0.041	1.141 (1.005, 1.296)	0.042	1.062 (0.901, 1.252)	0.472	1.125 (1.000, 1.265)	0.049
Threonate	IS	0.783 (0.617, 0.994)	0.044	0.787 (0.616, 1.006)	0.056	0.868 (0.621, 1.214)	0.409	0.775 (0.601, 1.000)	0.049
Tyrosine	ICH	5.908 (1.178, 29.641)	0.031	6.400 (1.210, 33.860)	0.029	19.397 (1.799, 209.111)	0.015	27.563 (0.040, 18,891.070)	0.023
7-Hoca	SAH	0.091 (0.013, 0.659)	0.018	0.084 (0.011, 0.663)	0.019	0.181 (0.011, 3.043)	0.235	0.280 (0.048, 1.645)	0.159
Choline	SAH	0.020 (0.001, 0.367)	0.008	0.022 (0.001, 0.362)	0.008	0.044 (0.001, 2.247)	0.120	0.000 (0.000, 0.125)	0.004
Glycine	SAH	0.343 (0.147, 0.800)	0.013	0.355 (0.151, 0.836)	0.018	0.404 (0.145, 1.122)	0.082	0.326 (0.140, 0.758)	0.009

MR, Mendelian randomization; CVD, cerebrovascular disease; OR, odds ratio; CI, confidence interval; MR.RAPS, Mendelian randomization robust adjusted profile score; IS, ischemic stroke; ICH, intracerebral hemorrhage; SAH, subarachnoid hemorrhage.

**Table 5:** Multivariable MR analyses of gut microbiota-derived metabolites on CVD after adjusting confounding factors.

Exposure	Outcome	Alcohol drinking		Smoking		Hypertension	
		OR (95% CI)	*P*	OR (95% CI)	*P*	OR (95% CI)	*P*
Cholesterol	IS	1.666 (1.091, 2.544)	0.018	1.621 (1.044, 2.517)	0.031	1.394 (0.921, 2.110)	0.116
Glycerate	IS	0.566 (0.312, 1.025)	0.060	0.597 (0.321, 1.108)	0.102	0.760 (0.418, 1.382)	0.369
Indolelactate	IS	0.654 (0.443, 0.967)	0.033	1.260 (0.849, 1.871)	0.251	0.642 (0.460, 0.897)	0.009
Leucine	IS	1.228 (0.861, 1.751)	0.257	1.267 (0.881, 1.821)	0.202	1.284 (0.905, 1.823)	0.162
Ornithine	IS	0.547 (0.326, 0.916)	0.022	0.527 (0.348, 0.798)	0.002	0.548 (0.311, 0.967)	0.038
Taurodeoxycholate	IS	1.134 (1.049, 1.225)	0.001	1.132 (1.034, 1.239)	0.008	1.125 (1.030, 1.229)	0.009
Threonate	IS	0.817 (0.672, 0.994)	0.043	0.809 (0.614, 1.066)	0.131	0.820 (0.654, 1.028)	0.085
Tyrosine	ICH	3.352 (0.550, 20.426)	0.190	2.275 (0.393, 13.151)	0.359	2.146 (0.369, 12.473)	0.395
7-Hoca	SAH	0.331 (0.033, 3.331)	0.348	0.299 (0.038, 2.341)	0.250	0.229 (0.024, 2.224)	0.204
Choline	SAH	0.050 (0.004, 0.695)	0.026	0.035 (0.003, 0.491)	0.013	0.016 (0.001, 0.183)	0.001
Glycine	SAH	0.370 (0.152, 0.896)	0.028	0.320 (0.074, 1.380)	0.126	0.216 (0.062, 0.754)	0.016

Multivariable MR (MVMR) analyses were conducted by using the inverse variance-weighted (IVW) method.

MR, Mendelian randomization; CVD, cerebrovascular disease; OR, odds ratio; CI, confidence interval; IS, ischemic stroke; ICH, intracerebral hemorrhage; SAH, subarachnoid hemorrhage.

## Discussion

Previous studies suggested that the gut microbiome exhibits high heritability and plays a pivotal role in cardiovascular disease (Kurilshikov et al., 2021; Zou et al., 2022), emphasizing the need for conducting microbial genome-wide association studies (mGWAS) in CVD. In the present study, we performed MR analyses to explore the causal relationship between gut microbiota, gut microbiota-derived metabolites, and CVD. Using summary data from the largest and latest GWAS, we detected the causal associations between 13 gut microbial genera and CVD subtypes. Moreover, it was suggested that the increased concentration of 11 metabolites was potentially protective or risk factors for different CVD subtypes, respectively.

As for gut microbiome and IS, a previous MR study reported that the bacterial genera *Intestinimonas* and *LachnospiraceaeNK4A136group* play significant protective roles in more than one IS subtype (Meng et al., 2023), which supported our results. Consistent with other previous observational studies (Denes et al., 2014; Huang et al., 2019; Ling et al., 2020a), *Streptococcus* was demonstrated to be related to a higher risk of IS in this study. *In-vitro* experiments also found that *Streptococcus* infection promoted atherosclerosis and aggravated ischemic brain damage through platelet and IL-1-mediated systemic inflammation (Denes et al., 2014). In a population-based study, Zeng et al. observed significantly lower levels of butyrate-producing bacteria *Lachnospiraceae* and *Ruminococcaceae* in the high-risk stroke group (Zeng et al., 2019), which was in line with our study. It is noteworthy that our results indicate varying effects of *LachnospiraceaeNK4A136group*, *LachnospiraceaeFCS020group*, and *LachnospiraceaeUCG010* on IS and ICH. This highlights the importance of conducting studies at a more specific species level and across different CVD subtypes to elucidate the potential mechanisms from the perspective of gut microbiota. As for ICH, a mouse model demonstrated a notably higher abundance of *Butyricimonas* in the exercise group (Liu et al., 2017). Regular exercise is known to reduce the risk of ICH (Carpenter et al., 2016), which supported our result that *Butyricimonas* exhibits a protective effect on the risk of ICH. Our findings indicated that *Catenibacterium* is associated with an elevated risk of ICH. Multiple observational studies have reported increased levels of *Catenibacterium* in obese patients (Gallardo-Becerra et al., 2020; Pinart et al., 2021). Meanwhile, a multicenter case–control study demonstrated that obesity can increase the risk of ICH (Pezzini et al., 2013). This suggests that *Catenibacterium* might influence ICH through the pathway of obesity. As for SAH, a previous study observed an increase in the relative abundance of *Enterorhabdus* following a low-calorie Mediterranean diet intervention (Pagliai et al., 2020). The Mediterranean diet is advised for preventing the development and rupture of cerebral aneurysms, which accounts for 85% of SAH cases (Czekajlo, 2019). The evidence above showed that *Enterorhabdus* may contribute to SAH risk with their function to the rupture of cerebral aneurysms.

Regarding the connection between gut microbiota-derived metabolites and CVD, consistent with our findings, a rat experiment using middle cerebral artery occlusion as the model confirmed the protective effect of ornithine against IS (Barakat et al., 2018). The NLRP3 inflammasome, characterized by leucine-rich repeat (LRR) domains at the C-terminus, promotes the initiation of an inflammatory response (Schroder and Tschopp, 2010). An experimental mouse model confirmed that NLRP3 inflammasome activation in neurons triggers neuroinflammation during acute IS. Early inhibition of NLRP3 reduces inflammation and stabilizes the blood–brain barrier, providing protection against ischemia/reperfusion injury (Franke et al., 2021). The evidence presented above supports our study’s conclusion that leucine is a risk factor for IS. Nagata et al. conducted a clinical trial to compare the cerebrospinal fluid of 6 aneurysmal SAH patients with 11 healthy controls and observed a higher concentration of 7-Hoca in patients (Nagata et al., 1995), which contradicted our findings. The limited sample size and sample heterogeneity are likely responsible for the conflicting results.

This study has several advantages. First, this study is the first to evaluate the causality between gut microbiota, gut microbiota-derived metabolites, and CVD. Second, the application of the MR method decreased the interference of confounding factors and the reverse causality of the results. Finally, based on the dataset from the largest GWAS up-to-date, we performed reverse MR, MVMR, and several sensitivity analyses to support the results. However, it has to be admitted that there are some limitations. First, the results should be interpreted with caution due to the inadequate IVs under genome-wide significance, which is why we utilized a loose cutoff for exposure-related SNPs at a threshold of *P*< 1 × 10^−5^. Second, bacterial taxa were only analyzed at the genus level rather than at a more specific species level. Finally, previous studies have demonstrated the contentious nature of gene–microbial interactions across diverse ethnicities. Therefore, the findings of this study may not be fully generalizable to other ethnic populations, given that the original GWAS primarily enrolled individuals of European descent.

In conclusion, by performing the two-sample MR analysis, we assessed the potential causality between gut microbiota, gut microbiota-derived metabolites, and CVD. Our findings could offer new perspectives on novel biomarkers for the targeted prevention and treatment of CVD. However, further randomized clinical trials and functional experimental studies are required to verify these findings and clarify the potential mechanism.

## Data availability statement

The original contributions presented in the study are included in the article/**Supplementary Material**. Further inquiries can be directed to the corresponding author.

## Author contributions

DL: Investigation, Writing – original draft. YZ: Writing – review & editing. ZT: Writing – review & editing. YT: Formal Analysis. CL: Formal Analysis. XP: Software, Visualization. JL: Software, Funding acquisition. XW: Writing – review & editing, Conceptualization, Funding acquisition.
