# Supplementary material for: Causal associations between gut microbiota, gut microbiota-derived metabolites, and cerebrovascular diseases: a multivariable Mendelian randomization study
Source: Front Cell Infect Microbiol. 2023 Nov 8;13:1269414. doi: 10.3389/fcimb.2023.1269414 (PMC10663354; doi:10.3389/fcimb.2023.1269414)
Supplement: Supplementary file 1 [file DataSheet_1.docx]

**Supplementary Figures 1-6**

**Causal Associations between Gut Microbiota, Gut Microbiota-derived Metabolites, and Cerebrovascular Diseases: A Multivariable Mendelian Randomization Study**

**Figure S1.** Leave-one-out plots for the causal association between gut microbiota and IS in forward MR analyses.

**Figure S2.** Leave-one-out plots for the causal association between gut microbiota and ICH in forward MR analyses.

**Figure S3.** Leave-one-out plots for the causal association between gut microbiota and SAH in forward MR analyses.

**Figure S4.** Leave-one-out plots for the causal association between gut microbiota-derived metabolites and IS in forward MR analyses.

**Figure S5.** Leave-one-out plots for the causal association between gut microbiota-derived metabolites and ICH in forward MR analyses.

**Figure S6.** Leave-one-out plots for the causal association between gut microbiota-derived metabolites and SAH in forward MR analyses.


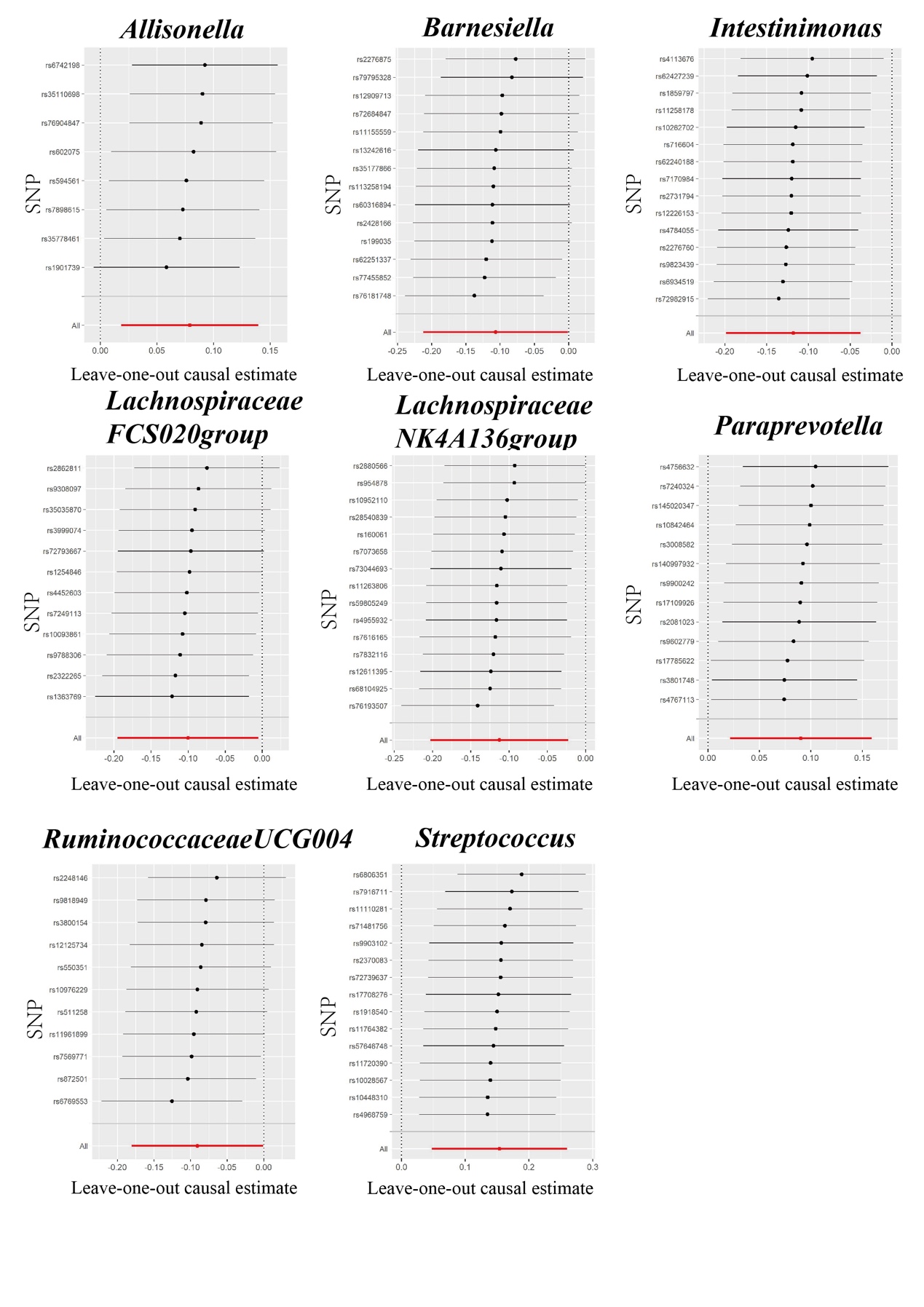


**Figure S1.** Leave-one-out plots for the causal association between gut microbiota and IS in forward MR analyses. The SNP in the figure represents the SNP sites involved in this MR analysis, and the horizontal black line represents 95% *CI* of the estimated causal effect value. Each black dot in the *CI* of each SNP is represented the effect value of MR causal estimation obtained when removing this SNP, using the remaining SNPs as IVs. IS, ischemic stroke; MR, mendelian randomization; SNP, single nucleotide polymorphism; *CI*, confidence interval; IVs, instrumental variables.


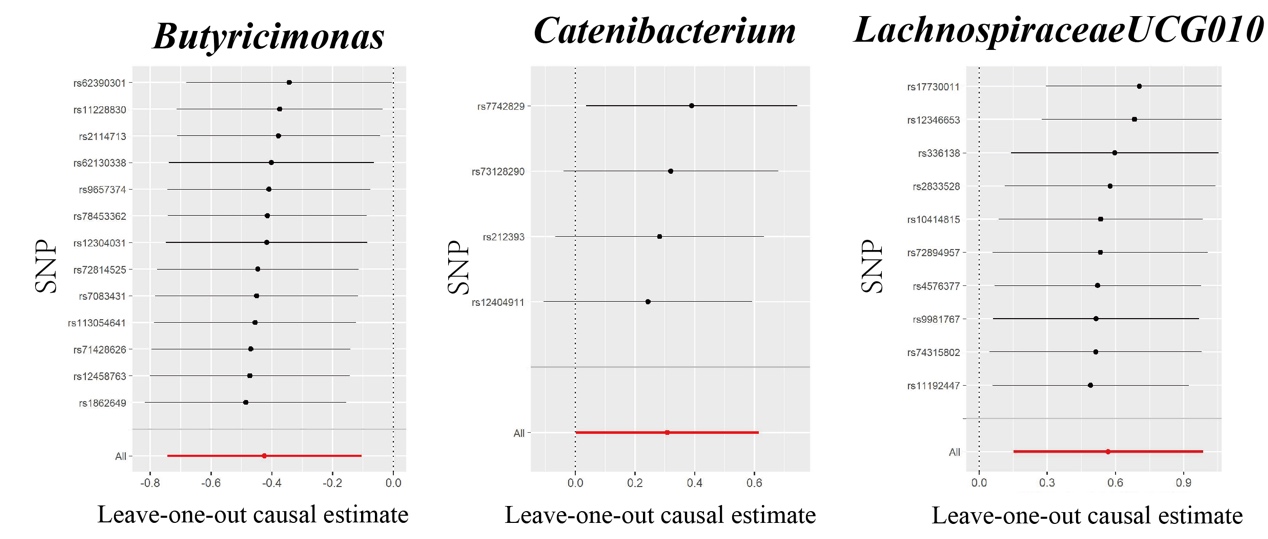


**Figure S2.** Leave-one-out plots for the causal association between gut microbiota and ICH in forward MR analyses. The SNP in the figure represents the SNP sites involved in this MR analysis, and the horizontal black line represents 95% *CI* of the estimated causal effect value. Each black dot in the *CI* of each SNP is represented the effect value of MR causal estimation obtained when removing this SNP, using the remaining SNPs as IVs. ICH, intracerebral hemorrhage; MR, mendelian randomization; SNP, single nucleotide polymorphism; *CI*, confidence interval; IVs, instrumental variables.


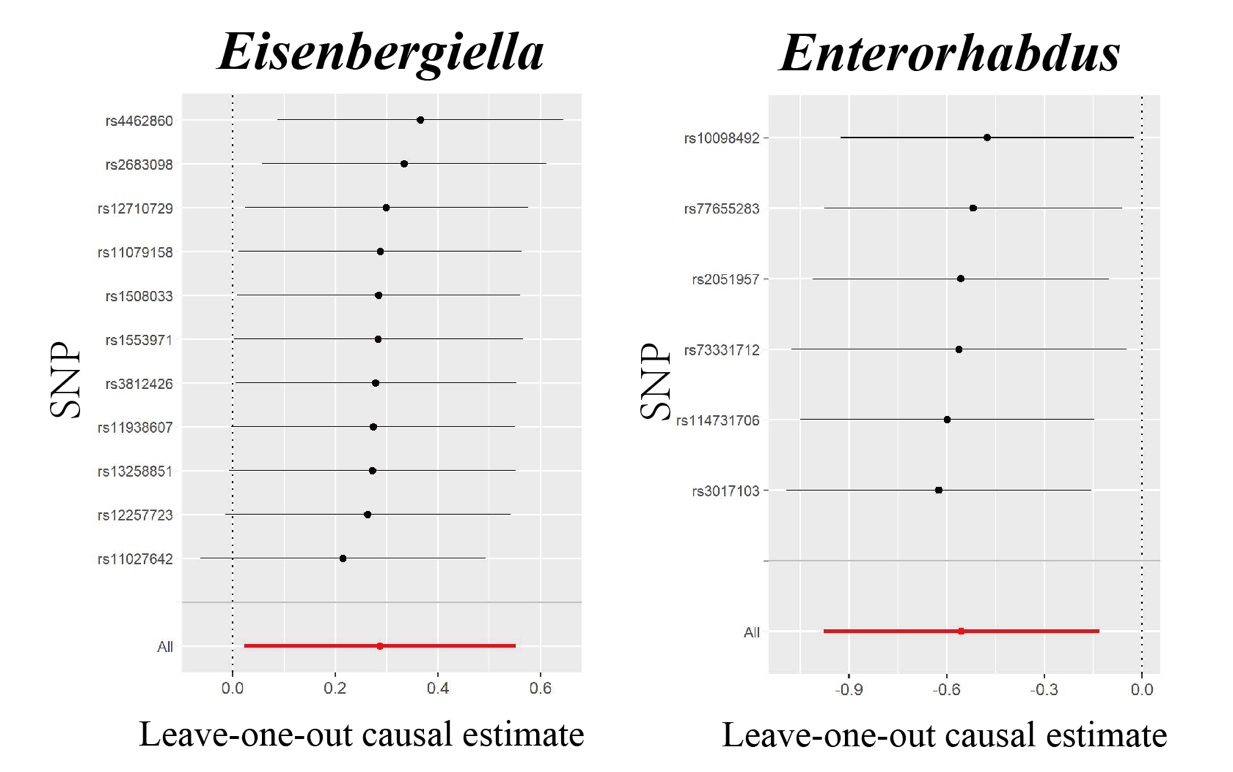


**Figure S3.** Leave-one-out plots for the causal association between gut microbiota and SAH in forward MR analyses. The SNP in the figure represents the SNP sites involved in this MR analysis, and the horizontal black line represents 95% *CI* of the estimated causal effect value. Each black dot in the *CI* of each SNP is represented the effect value of MR causal estimation obtained when removing this SNP, using the remaining SNPs as IVs. SAH, subarachnoid hemorrhage; MR, mendelian randomization; SNP, single nucleotide polymorphism; *CI*, confidence interval; IVs, instrumental variables.


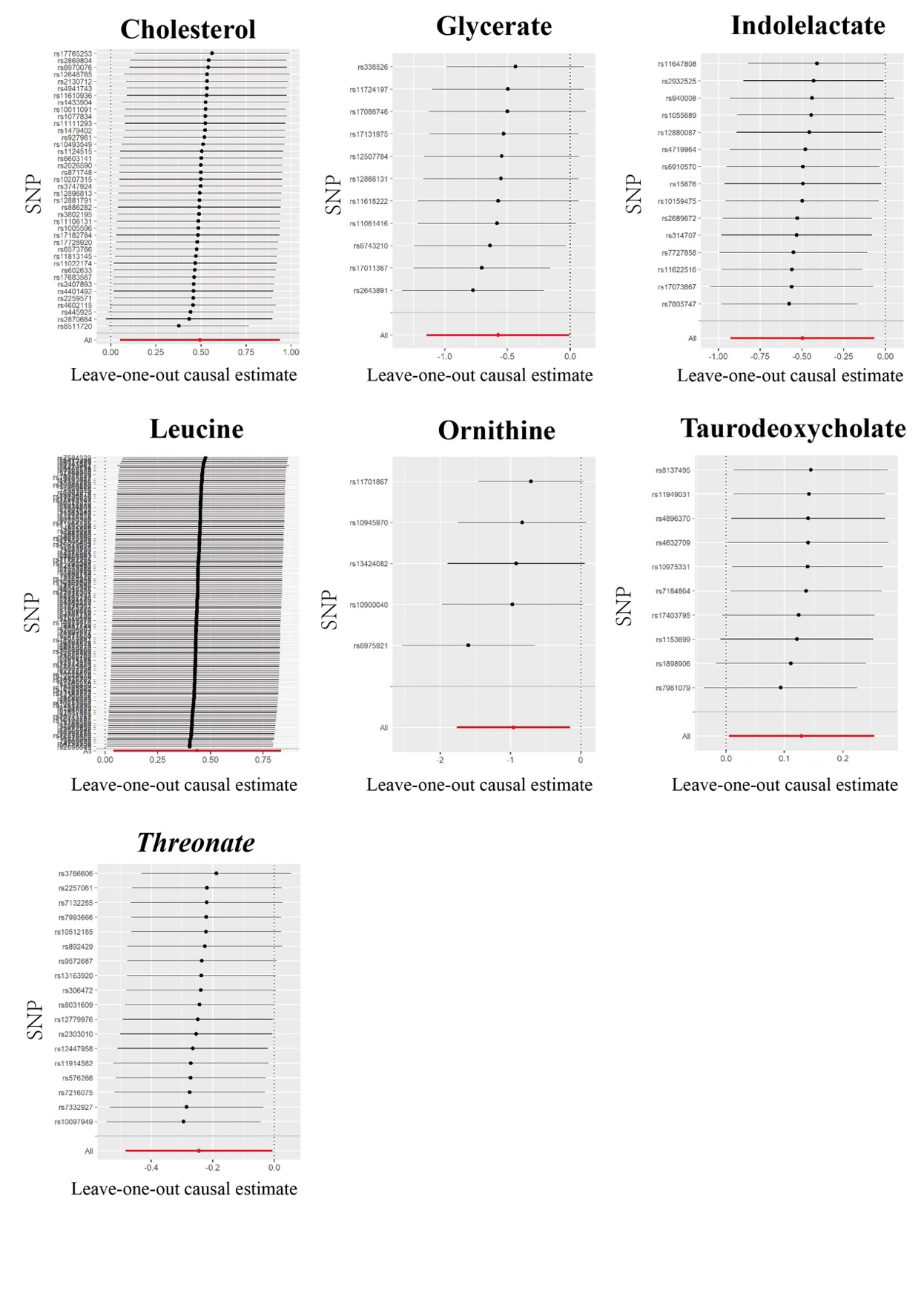


**Figure S4.** Leave-one-out plots for the causal association between gut microbiota-derived metabolites and IS in forward MR analyses. The SNP in the figure represents the SNP sites involved in this MR analysis, and the horizontal black line represents 95% *CI* of the estimated causal effect value. Each black dot in the *CI* of each SNP is represented the effect value of MR causal estimation obtained when removing this SNP, using the remaining SNPs as IVs. IS, ischemic stroke; MR, mendelian randomization; SNP, single nucleotide polymorphism; *CI*, confidence interval; IVs, instrumental variables.


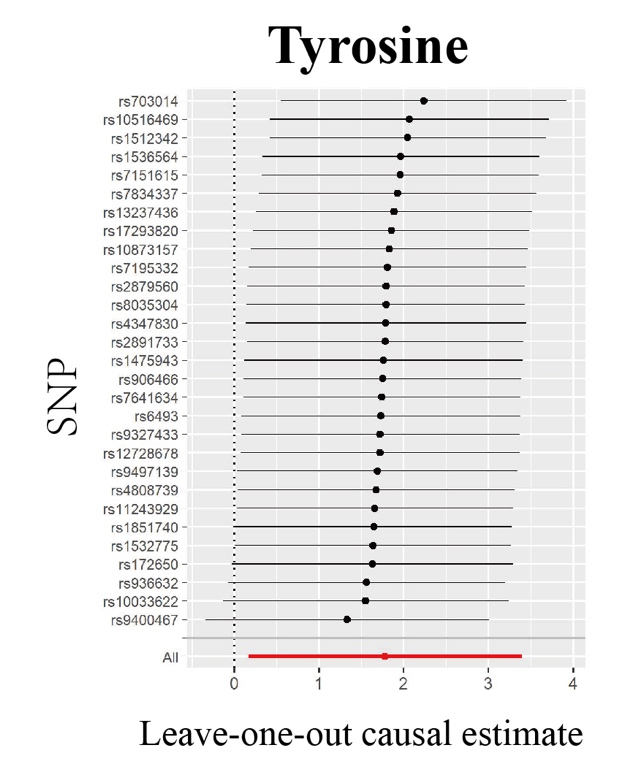


**Figure S5.** Leave-one-out plots for the causal association between gut microbiota-derived metabolites and ICH in forward MR analyses. The SNP in the figure represents the SNP sites involved in this MR analysis, and the horizontal black line represents 95% *CI* of the estimated causal effect value. Each black dot in the *CI* of each SNP is represented the effect value of MR causal estimation obtained when removing this SNP, using the remaining SNPs as IVs. ICH, intracerebral hemorrhage; MR, mendelian randomization; SNP, single nucleotide polymorphism; *CI*, confidence interval; IVs, instrumental variables.


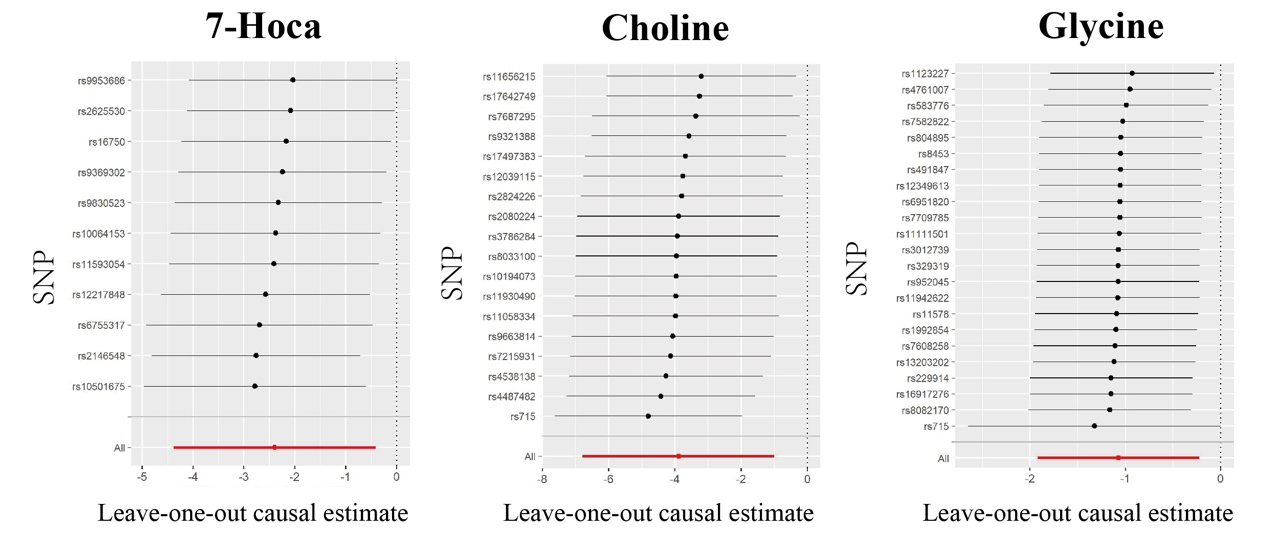


**Figure S6.** Leave-one-out plots for the causal association between gut microbiota-derived metabolites and SAH in forward MR analyses. The SNP in the figure represents the SNP sites involved in this MR analysis, and the horizontal black line represents 95% *CI* of the estimated causal effect value. Each black dot in the *CI* of each SNP is represented the effect value of MR causal estimation obtained when removing this SNP, using the remaining SNPs as IVs. SAH, subarachnoid hemorrhage; MR, mendelian randomization; SNP, single nucleotide polymorphism; *CI*, confidence interval; IVs, instrumental variables.
